# An anatomical investigation of alkaptonuria: Novel insights into ochronosis of cartilage and bone

**DOI:** 10.1111/joa.14190

**Published:** 2024-12-20

**Authors:** Juliette H. Hughes, Gemma Charlesworth, Amanda Prior, Claire M. Tierney, Paul D. Rothwell, Neil P. Thomas, Lakshminarayan R. Ranganath, James A. Gallagher, Alistair P. Bond

**Affiliations:** ^1^ Department of Musculoskeletal and Ageing Science, Institute of Life Course and Medical Science University of Liverpool Liverpool UK; ^2^ Liverpool Shared Research Facilities, Faculty of Health and Life Sciences University of Liverpool Liverpool UK; ^3^ Human Anatomy Resource Centre, Education Directorate University of Liverpool Liverpool UK; ^4^ Department of Clinical Biochemistry and Metabolic Medicine Royal Liverpool University Hospital Liverpool UK

**Keywords:** alkaptonuria, bone, cartilage, connective tissue, ochronosis, perichondrium, pigmentation

## Abstract

Ochronotic pigmentation of connective tissue is the central pathological process in the rare metabolic disease alkaptonuria (AKU). Tissue pigmentation in AKU occurs due to unmetabolised homogentisic acid (HGA) in the circulation, caused by an enzyme deficiency in the liver. Ochronotic pigmentation, derived from HGA, has previously been reported and described in large joints obtained from arthroplasty surgeries, which typically have advanced disease. Many tissues that are affected by ochronosis are not accessible for study during life, including tissues subjected to early and mid‐stage disease. Here, the opportunity arose to anatomically examine a 60‐year‐old AKU female body donor, allowing the investigation of previously understudied tissue, including those undergoing early‐stage pathological changes. Dissection of fresh‐frozen tissue was carried out and harvested tissues were fixed and examined histologically using H&E and Schmorl's stains to aid identification of ochronotic pigment. This work focusses on osteochondral tissues including extra‐skeletal cartilage, viscera and eyes. Gross and histological images demonstrating pigmentation in the cartilage and perichondrium of the ear ossicles, tympanic membrane and the pubic symphysis fibrocartilaginous disc are described for the first time here. We also show the first examination of the temporomandibular joint, which macroscopically appeared unpigmented, with histological analysis of the fibrocartilaginous disc showing no pigmentation. Pigmentation of non‐articular hyaline cartilage was observed in the respiratory tract, in both the hyaline cartilage and perichondrium, confirming previous findings. Within smaller joints, pigmentation of chondrons and the surrounding territorial matrix was observed, but was confined to calcified articular cartilage, and was not generally found in the hyaline articular cartilage. Dark pigmentation of the perichondrium adjacent to the articular surface was observed in numerous small joints. The calcified bone matrix was not pigmented but ochronosis was identified in a small fraction of trabecular osteocytes in the capitate and radius, with substantially more pigmented osteocytes observed in bone of the ear ossicles. Viscera examined were unpigmented. This anatomical examination of tissues from an AKU individual highlights that most osteochondral tissues are susceptible to HGA‐derived pigmentation, including the ear ossicles which are the smallest bones in the body. Within joints, calcified cartilage and perichondrium appear to be the earliest affected tissues, but why this is the case is not understood. Furthermore, why the TMJ disc was unaffected by pigmentation is intriguing. The heterogenous appearance of pigmentation both within and between different tissues indicates that factors other than tissue type (i.e. cartilage, perichondrium) and matrix composition (i.e. collagen‐rich, calcified) may affect the process of ochronosis, such as oxygen tension, loading patterns and tissue turnover. The effect of nitisinone treatment on the ochronotic disease state is considered, in this case 7 years of treatment, however comparisons could not be made to other cases due to inter‐individual variability.

## Graphical abstract

Examination of an alkaptonuria body highlights the susceptibility of all cartilage types and associated perichondrium to ochronotic pigmentation and the heterogeneity of ochronotic pigment distribution both within and between tissues. In joints, calcified cartilage pigments before non‐calcified cartilage. Perichondrial pigmentation precedes hyaline cartilage of non‐skeletal cartilages. Previously unexamined tissues such as the ear ossicles, TMJ disc and sclera are reported.

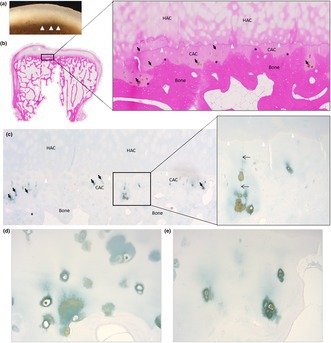

## 1 INTRODUCTION

Alkaptonuria (AKU; OMIM #203500) is an ultra‐rare metabolic disease that results in degeneration and failure of connective tissues (Phornphutkul et al., 2002). Autosomal recessive mutations in the homogentisate 1,2‐dioxygenase (HGD) gene lead to an inability to breakdown homogentisic acid (HGA) in AKU (Fernández‐Cañón et al., 1996; La Du et al., 1958; Zatkova et al., 2012). Unmetabolised HGA is excreted into the urine, but it still elevated in the circulation and tissue fluid. The deposition of a yellow (or ‘ochre’) to dark brown/black pigment into connective tissues, a process called ochronosis, is characteristic of AKU and ultimately causes devastating joint destruction and connective tissue disorder (O'Brien et al., 1963; Virchow, 1866), and heart valve disease (Hannoush et al., 2012; Helliwell et al., 2008; Phornphutkul et al., 2002). In early adulthood, backpain and joint pain begin as early as the third decade (Cox et al., 2019), progressing to a severe and early‐onset osteoarthropathy of the spine and large joints, resulting in joint replacement and immobility of the spine (Mannoni et al., 2004; Phornphutkul et al., 2002). Other affected tissues include tendons and ligaments leading to rupture, and the heart valves causing stenosis. Collectively these pathological changes contribute to deteriorating quality of life.

Ochronosis of connective tissue, such as the sclera of the eye and cartilage of the ear, typically takes two to three decades to become visible externally, with ear cartilage biopsies from adult AKU patients detecting pigmentation at the age of 20 years (Cox et al., 2019). However, a recent paediatric study reported scleral pigmentation in three out of 13 AKU children, with the earliest observed at 13 years, with no externally visible pigmentation of ear cartilage (Kujawa et al., 2023). Due to the location of articular cartilage and other joint tissues, they cannot easily be studied, particularly at the microscopic level, to determine when pigment is first deposited. Despite the rarity of AKU, the ochronotic phenotype of AKU joints donated after surgery has been documented to some degree, offering valuable insights into the disease process within articular cartilage (Boyde et al., 2014; Chow et al., 2020; Taylor et al., 2011, 2017, 2019). Other tissues are either not replaceable or do not cause severe enough symptoms to warrant replacement and are therefore unavailable to study (i.e. spine, respiratory cartilage). Other than invasive examinations, such as arthroscopy and biopsy, which require medical reasons to carry out such procedures, surgical insights and post‐mortems are the only other opportunities to investigate ochronosis and related pathologies.

Many surgical case reports exist in the literature, often coupled with a brief history of the patient and radiographical findings and symptoms. Examples include bronchoscopy (Parambil et al., 2005), meniscus rupture and arthroscopy (Nag et al., 2013; Xu et al., 2015), arthroplasty (Cebesoy et al., 2014; Khalifa et al., 2022; Merolla et al., 2012) and tendon/ligament rupture (Jiang et al., 2019; Manoj Kumar & Rajasekaran, 2003; Mwafi et al., 2021). Such case reports vary in length and detail, with some showing gross photographs, with even fewer showing histology. Detailed descriptions of AKU patients both antemortem and post‐mortem published approximately 70 years ago document individuals with severe disease but lack both high‐quality gross photographs and clear, high‐resolution histology photomicrographs (Galdston et al., 1952; Lichtenstein & Kaplan, 1953). A more recent post‐mortem examination of an AKU patient was carried out approximately 15 years ago, providing the first modern gross and histological insight into the appearance of tissue ochronosis (Helliwell et al., 2008). Overall, the available literature provides a good summary of the location of ochronotic pigment across different body tissues at the macroscopic level, with more investigation into tissue ochronosis needed at the microscopic level to gain further insight into the disease process.

Currently, connective tissue disorder in AKU can only be treated by palliative strategies such as pain management, total joint arthroplasty, ligament and tendon repair after rupture and heart valve replacement (Ranganath et al., 2013). None of these treatments intervene in the pathophysiological process of ochronosis and subsequent biomechanical failure of tissues, but intervene at the point of, or after, failure of the tissue. The process of ochronosis is not well understood, and neither are the changes that lead to tissue dysfunction. It is not known where in the extracellular matrix HGA‐derived pigment binds, nor has the chemical composition of pigmentation been identified (Ranganath et al., 2019). AKU tissue ages and degenerates rapidly, even quicker than the general osteoarthritic population, with a three times greater incidence of joint replacement in AKU than in osteoarthritis (OA) (Ranganath et al., 2021). Understanding AKU pathophysiology may be the key towards development of therapies that prevent tissue degeneration in AKU, which also may be applicable to other general, degenerative diseases such as OA, tendinopathy and valve stenosis, where common mechanisms may exist that are amplified by AKU. If HGA‐derived pigment can bind selectively to specific connective tissues and cause degeneration, it is likely that other small molecules do the same that cannot be seen due to not forming a visible pigment. Even though a surprising number of papers/case reports over many years have documented pigmented tissues in AKU, most have only been able to grossly describe the location of pigmentation and the clinical signs of tissue pathology that result. Determining where HGA‐derived pigment binds to specific tissues, and the order of pigmentation with tissue ageing, is key to understanding the process not just of AKU arthropathy, but also other degenerative diseases, such as OA.

Here, we were provided with an exceptionally rare opportunity to examine tissues from an AKU body donor. Anatomical and histological examination of the organs and tissues was carried out to describe the distribution of ochronotic pigmentation and identify any related pathology with a focus on osteochondral tissue, including previously unexamined tissues (i.e. ear ossicles, tympanic membrane, temporomandibular joint, small joint cartilage and sclera). Previous replacement of six large joints due to AKU arthropathy in this individual suggested that we would find evidence of severe ochronosis in other joints and tissues. Whilst intense pigmentation was observed at some anatomical sites such as the costal cartilage, others were much less pigmented, allowing examination of tissues in the early stages of ochronosis.

## 2 MATERIALS AND METHODS

### 2.1 Ethical approval

A 60‐year‐old female with alkaptonuria donated her body post‐mortem to the University of Liverpool for anatomical examination. Ethical approval was obtained from the University of Liverpool Central Research Ethics Committee (reference number: 5785). Consent was obtained prior to death, which included the use of photographs for research purposes. A right femoral head from the same individual was obtained antemortem as surgical waste under ethical approval from the NHS Liverpool Central Research Ethics Committee (REC reference: 07/Q1505/29), with informed consent for use for research purposes, including photographs.

### 2.2 Donor information

The individual had been receiving nitisinone treatment (2 mg/daily) for 7 years. Prior to nitisinone treatment, serum HGA was 42.6 μmol/L and 24‐hour urinary HGA was 24.2 mmol/L. Following 72 months of nitisinone treatment (2 mg/daily) serum HGA decreased to <3.1 μmol/L and 24‐hour urinary HGA was 0.4 mmol/L.

### 2.3 Dissection

The donor was refrigerated for 7 days prior to arriving at University of Liverpool, when the body was placed in −20°C for storage. The body was defrosted in the refrigerator at 4°C, prior to dissection, which was carried out over 5 days in a regional manner. Photographs were taken of anatomical tissues and structures in situ and after removal. Smaller samples were removed for histological analysis, see Table 1. All tissue from the dissection and samples taken for analysis were disposed of following local departmental regulations in accordance with the Human Tissue Act (2004).

**TABLE 1:** Presence/absence of pigmentation in tissues taken for histological analysis. For processing protocols, see Supplementary Table S1.

Tissue	Left or right	Macroscopic pigmentation	Microscopic pigmentation	Processing protocol
Temporomandibular joint disc	Left	No	No	2
Malleus and tympanic membrane	Left	Yes	Yes	2
Incus	Left	No	Yes	2
Stapes	Left	No	Yes	2
Pubic symphysis joint	N/A (midline)	No	Yes	2
Costal cartilage of second sternocostal junction	Left	Yes	Yes	2
Auricle cartilage (conchal bowl)	Left	No	Yes	2
Auricle cartilage (conchal bowl)	Right	Yes	Yes	2
External acoustic meatus	Right	Yes	Yes	2
Nasal septum	N/A (midline)	Yes	Yes	2
Tracheal ring (superior mediastinum)	N/A (midline)	Yes	Yes	2
Primary bronchus cartilage	Left	Not examined	Yes	2
Secondary bronchus cartilage	Left	Not examined	Yes	2
Radial head	Left	Yes	Yes	2
Capitate	Right	Yes	Yes	2
Fifth distal interphalangeal joint	Right	No	Yes	2
Lateral cuneiform	Left	Yes	Yes	2
Sclera	Right	Yes	Yes	3
Sclera	Left	Yes	Yes	3
Oesophagus	N/A	No	No	1
Stomach	N/A	No	No	1
Duodenum	N/A	No	No	1
Jejunum	N/A	No	No	1
Ileum	N/A	No	No	1
Large intestine (descending colon)	N/A	No	No	1
Liver (left lobe)	N/A	No	No	1
Gallbladder	N/A	No	No	1
Pancreas	N/A	No	No	1
Spleen	N/A	No	No	1
Kidney	Left	No	No	1
Bladder	N/A	No	No	1
Ovary	Right	No	No	1
Gastrocnemius muscle (near Achilles)	Left	No	No	1
Parotid gland	Left	No	No	1
Sublingual gland	Left	No	No	1
Submandibular gland (deep part)	Left	No	No	1
Lacrimal gland	Right	No	No	1
Carotid sheath lymph node	Left	No	No	1
Tracheal Lymph node	N/A	No	No	1
Lung parenchyma (inferior lobe)	Right	No	No	1

*Note*: N/A not applicable.

### 2.4 Histology

Samples from dissected tissues were fixed in 10% phosphate buffered formalin for a minimum of 48 hours, then stored in 70% ethanol until processing. Tissues that contained mineral were decalcified prior to tissue processing, by immersion in Formical‐2000 (Statlab, US) until mineral was no longer present, which varied from several hours to several days with replenishment of the solution depending on the size of the tissue. Tissues were processed using a Leica ASP300 tissue processor, using Formula R paraffin wax (Leica, Germany). Three different processing protocols were used; all viscera using protocol 1, bone and cartilage using protocol 2 and sclera using protocol 3, see Supplementary Table S1 for protocol details. Tissues were embedded in Formula R paraffin wax and sectioned at 4.5–5 μm using a Leica RM2235 microtome and mounted on glass Superfrost® Plus microscope slides (ThermoScientific, UK).

Sections were stained for ochronotic pigment using a modified Schmorl's stain, which stains the yellow‐brown pigment a dark blue‐green colour (Hughes et al., 2021; Tinti et al., 2011). Briefly, sections were deparaffinised, rehydrated, incubated in Schmorl's stain (1% ferric chloride, 1% potassium ferricyanide in distilled water), immersed in 1% acetic acid and counterstained with nuclear fast red (full protocol in Supplementary Information). Sections were stained with haematoxylin and eosin (H&E) following deparaffination and rehydration, see Supplementary Information for full protocol. Slides were imaged using a Zeiss Axioscan Z1 slide scanner and processed using Zen 3.8 software (Zeiss).

## 3 RESULTS

Pigmentation of tissue typically appears as a black/brown colour grossly, and when sectioned, appears as a golden yellow or brown colour. H&E staining can mask less intense pigment. Schmorl's staining was therefore carried out alongside H&E, to stain the pigment a green‐blue colour, aiding its identification (Hughes et al., 2021; Preston et al., 2014; Taylor et al., 2012; Tinti et al., 2011).

### 3.1 Temporomandibular joint

Upon gross examination of the temporomandibular joints (TMJ), the articular surfaces of the mandibular fossa/articular tubercle of the temporal bone and the mandibular head of the mandible did not appear pigmented (right and left, not shown). The articular surfaces of the TMJ were not examined histologically. The articular disc of the left TMJ, which is composed of dense fibrocartilage and fibrous connective tissue (Detamore & Athanasiou, 2003; Runci Anastasi et al., 2021), appeared normal, both grossly and histologically, with no pigmentation identified with Schmorl's staining (Supplementary Figure S1). Note that fibrocartilage does not have a perichondrium.

### 3.2 Ear ossicles

**FIGURE 1:**
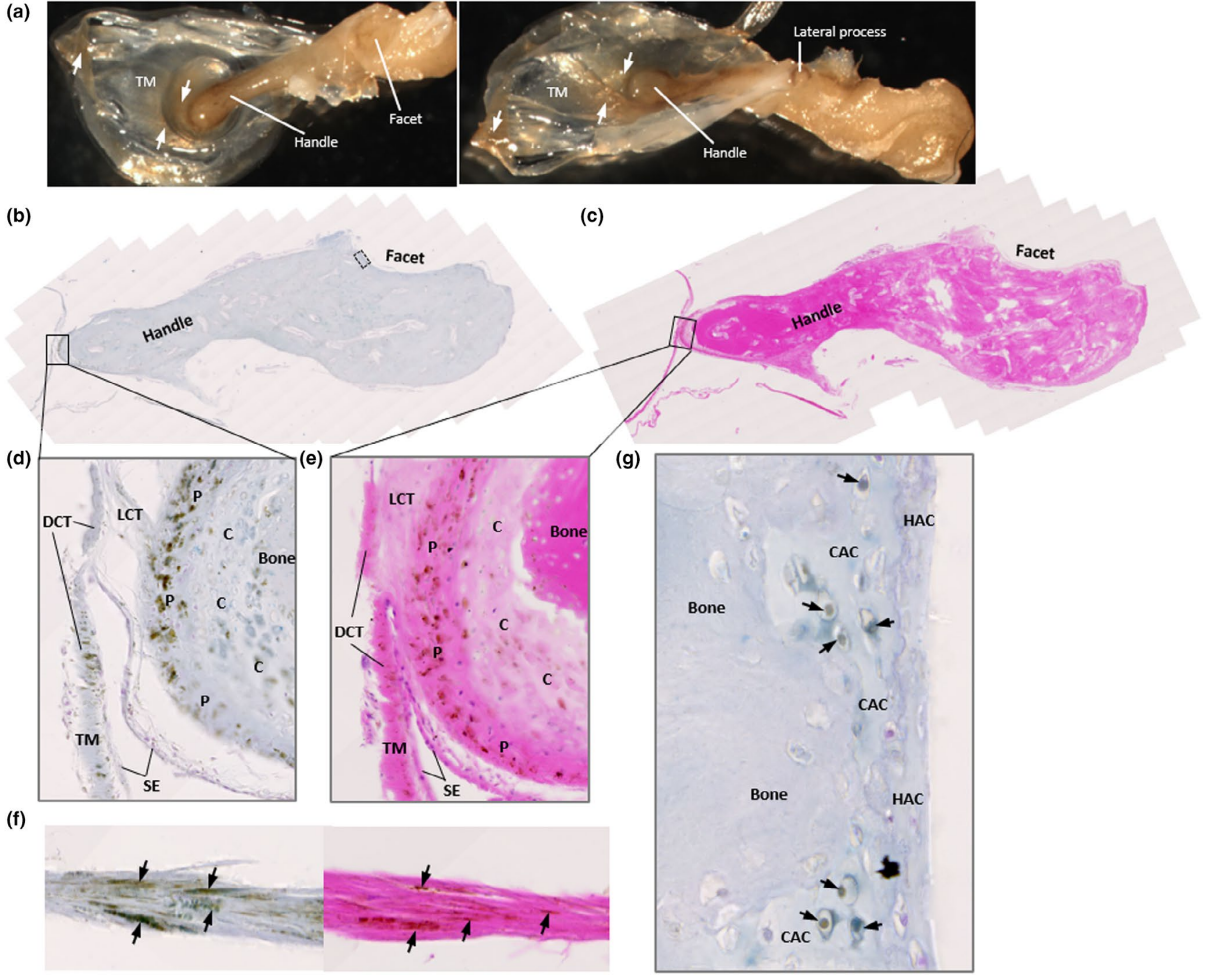
Ochronotic pigmentation of the malleus and tympanic membrane. The malleus is shown in (a) with the attached tympanic membrane (TM), which appears pigmented (white arrows) from the central attachment of the handle of the malleus, radiating outwards. The tympanic membrane also appears pigmented at the periphery. The lateral process of the malleus can be observed in the right image, and also appears pigmented. (b) Schmorl's staining and (c) H&E staining of the whole malleus sectioned longitudinally, including part of the TM at its attachment to the handle of the malleus and the facet for articulation with the incus. (d) and (e) higher magnification images of the attachment of the TM at the umbo/tip of the handle of the malleus. The TM is composed of a dense connective tissue (DCT) that appears pigmented. The tip of the handle of the malleus has been labelled to show the bone, cartilage (C), the perichondrium (P), with intense pigmentation observed in the perichondrium and the cartilage. Intervening between the DCT and perichondrium is loose connective tissue (LCT), which appears unpigmented. Lining both the TM and perichondrium is a layer of unpigmented, simple squamous epithelium (SE). (f) Schmorl's (left) and H&E (right) staining of the TM near to the lateral process of the malleus, where intense pigmentation is observed running in the direction of fibre orientation of the TM. (g) pigmented chondrons (black arrows) within the calcified articular cartilage (CAC) of the articular facet of the malleus from the inset area shown in (b), with no pigmented chondrons observed in the hyaline articular cartilage (HAC).

The tympanic membrane attached to the malleus was pigmented both grossly and histologically, particularly at its attachment to the handle of the malleus at the umbo and lateral process (Figure 1a–f), and also towards the periphery of the membrane. Pigmentation in places appeared to be orientated with fibre direction (Figure 1f). Figure 1d,e were annotated with reference to the histological images by De Greef et al. (De Greef et al., 2016) and Graham et al. (Graham et al., 1978), who investigated the connection between the tympanic membrane and malleus, termed the tympano‐mallear connection. The malleus was very pigmented where the tympanic membrane attaches (Figure 1d,e), with dark pigmentation in the perichondrium lining the cartilage that encases the bone forming the distal end of the malleus handle. Chondrocyte pigmentation was observed. Dense regular connective tissue (DCT) within the tympanic membrane could be observed, particularly with H&E staining (Figure 1e), in addition to loose connective tissue (LCT) pigmentation, that is not as eosinophilic as DCT. In the H&E stained tympano‐mallear connection, the DCT can be seen blending with the perichondrium. The DCT of the tympanic membrane was pigmented, whilst the LCT and the simple squamous epithelium was not. The articular facet of the malleus that articulates at the synovial incudo‐malleolar joint was grossly unpigmented (Figure 1a), however pigmented chondrons were observed histologically within the calcified articular cartilage (CAC) of the facet (Figure 1g). Most of these pigmented chondrons were deep to the tidemark, with a few pigmented chondrons observed superficially within the hyaline articular cartilage (HAC).

**FIGURE 2:**
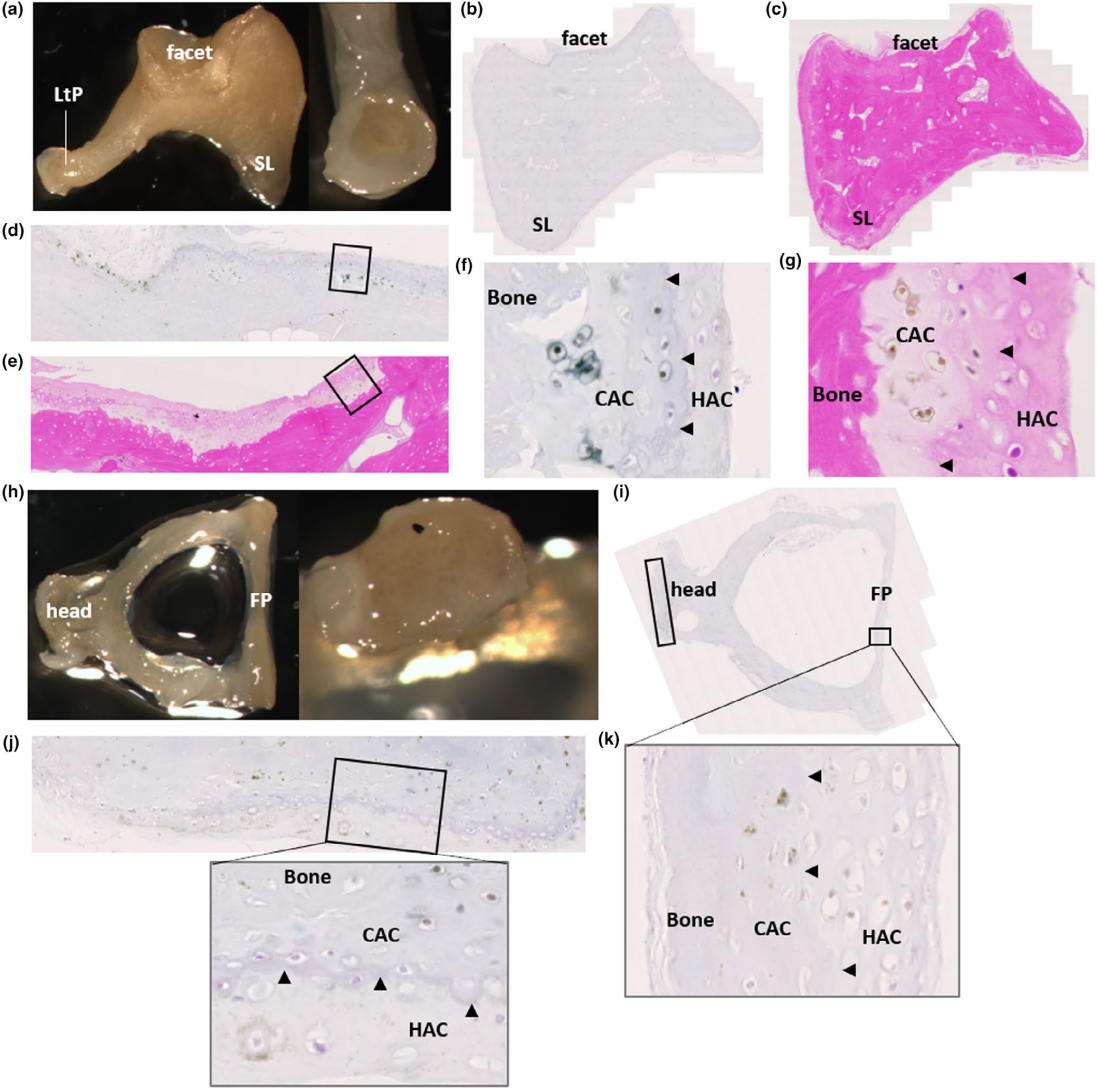
Ochronotic pigmentation of the incus and stapes. The incus is shown in (a), where the short limb (SL), lenticular process (LtP) and facet can be observed, with the right image showing the articular surface of the lenticular process (LtP). Macroscopically, no pigmentation was identified in the incus. (b) and (c) H&E and Schmorl's staining of the incus, with the articular facet for the malleus towards the top of the image and the short limb identified (SL). (d) and (e) the articular facet of the incus stained with Schmorl's and H&E, respectively, with the insets shown at higher magnification in (f) and (g). Pigmentation of chondrocytes within calcified articular cartilage (CAC) was observed, with no pigmentation observed in the hyaline articular cartilage (HAC), superficial to the tidemark (indicated by arrow heads). The stapes are shown in (h), with the head and footplate annotated, with the right image showing the distal end of the head of the stapes which appears unpigmented. (i) Schmorl's staining of the stapes, with higher magnification images of the insets shown in (j) and (k). (j) the head of the stapes, which articulates with the incus. Pigment can be observed in the CAC, and also in the HAC superficial to the tidemark (indicated by arrowheads), surrounding a chondrocyte. (k) the footplate of the stapes, which shows pigmentation of chondrons in the CAC (tidemark indicated with arrowheads).

Grossly, both the incus (Figure 2a) and stapes (Figure 2h) appeared normal. Histologically, pigmentation of the articular facet of the incus that articulates with the malleus was observed (Figure 2b–g), with pigmented chondrons observed in the CAC layer, and not the superficial HAC. The lenticular process of the incus was not examined histologically. The head of the stapes, that forms an articulation with the long limb of the incus at the incudostapedial joint, and the footplate (or base) of the stapes that rests on the oval window, can be observed in Figure 2h and histologically in Figure 2i–k, where pigmentation of chondrons within the CAC can be observed. In the footplate of the stapes, pigment was confined to CAC, and not present in the HAC or bone. In the head of the stapes, pigmentation surrounding a chondrocyte in HAC was observed.

**FIGURE 3:**
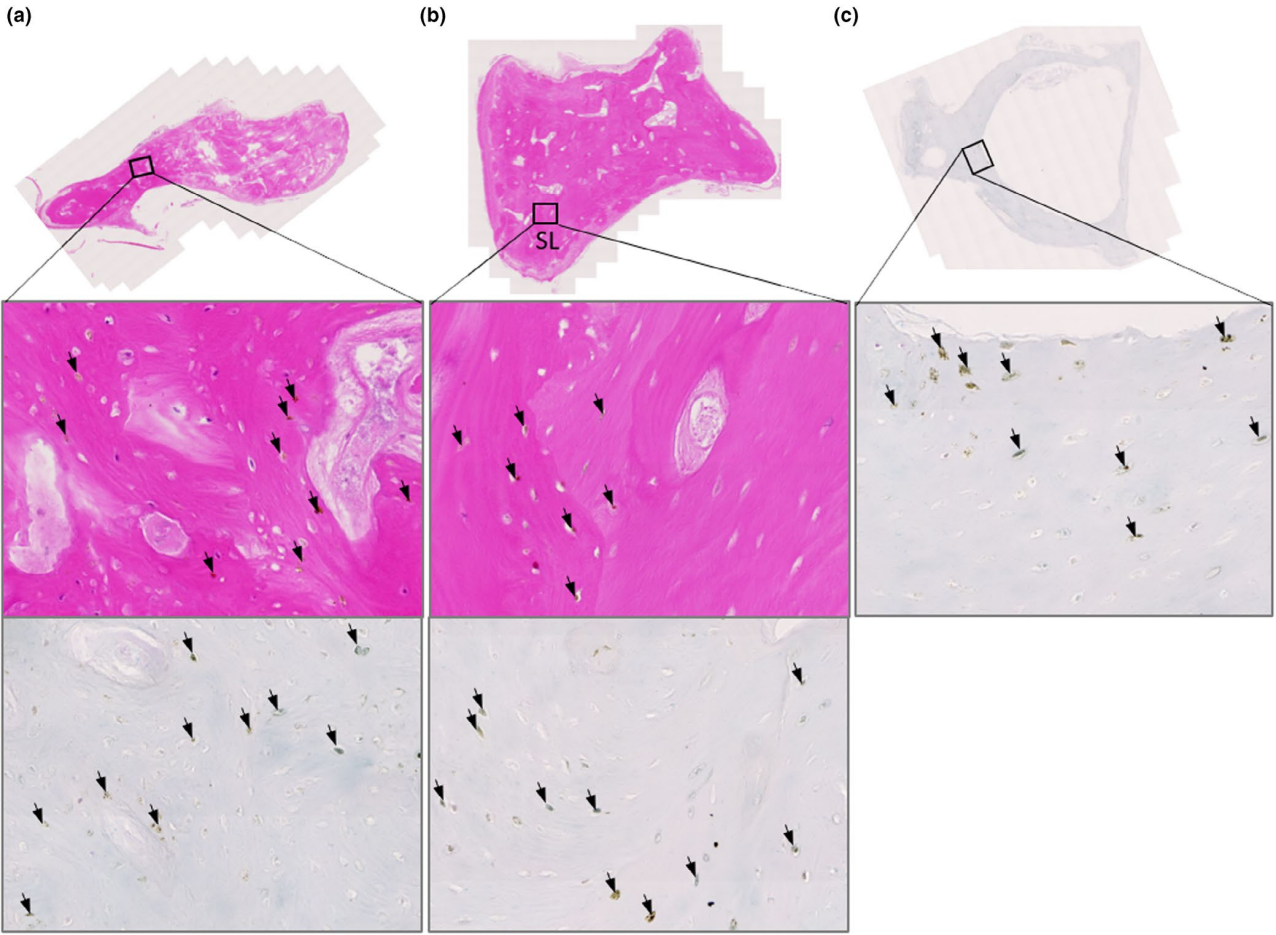
Ochronotic pigmentation of ear ossicle osteocytes. The malleus and incus stained with H&E in (a) and (b) respectively. The insets in (a) and (b) are shown below, with a corresponding area stained with Schmorl's. The stapes stained with Schmorl's in (c), with the inset shown below. Arrows indicate pigmented cells (osteocytes) within the bone, which can be observed with both H&E and Schmorl's staining.

When examining the ear ossicles, it was also observed that osteocytes within the bone were pigmented. Figure 3a–c shows an example of pigmented osteocytes in the malleus, incus and stapes, with both H&E staining and Schmorl's staining showing pigmented cells within the bone matrix (see arrows). Whilst many pigmented osteocytes were observed, there were still many non‐pigmented osteocytes.

### 3.3 Pubic symphysis

In a coronal cross section through the pubic symphysis (Supplementary Figure S1), the joint looked unpigmented, including the fibrocartilaginous disc. The superior part of the pubic symphysis examined histologically showed pigmentation within the disc that was intense and patchy, and associated with cells, assumed to be chondrocytes (Supplementary Figure S2b‐c). The disc had a cleft, which is considered a normal feature in some individuals (Becker et al., 2010). The thin hyaline cartilage layer showed some very light pigmentation of chondrocytes with Schmorl's staining (Supplementary Figure S2d), with very few pigmented cells observed. The bone appeared normal.

### 3.4 Costal cartilage

**FIGURE 4:**
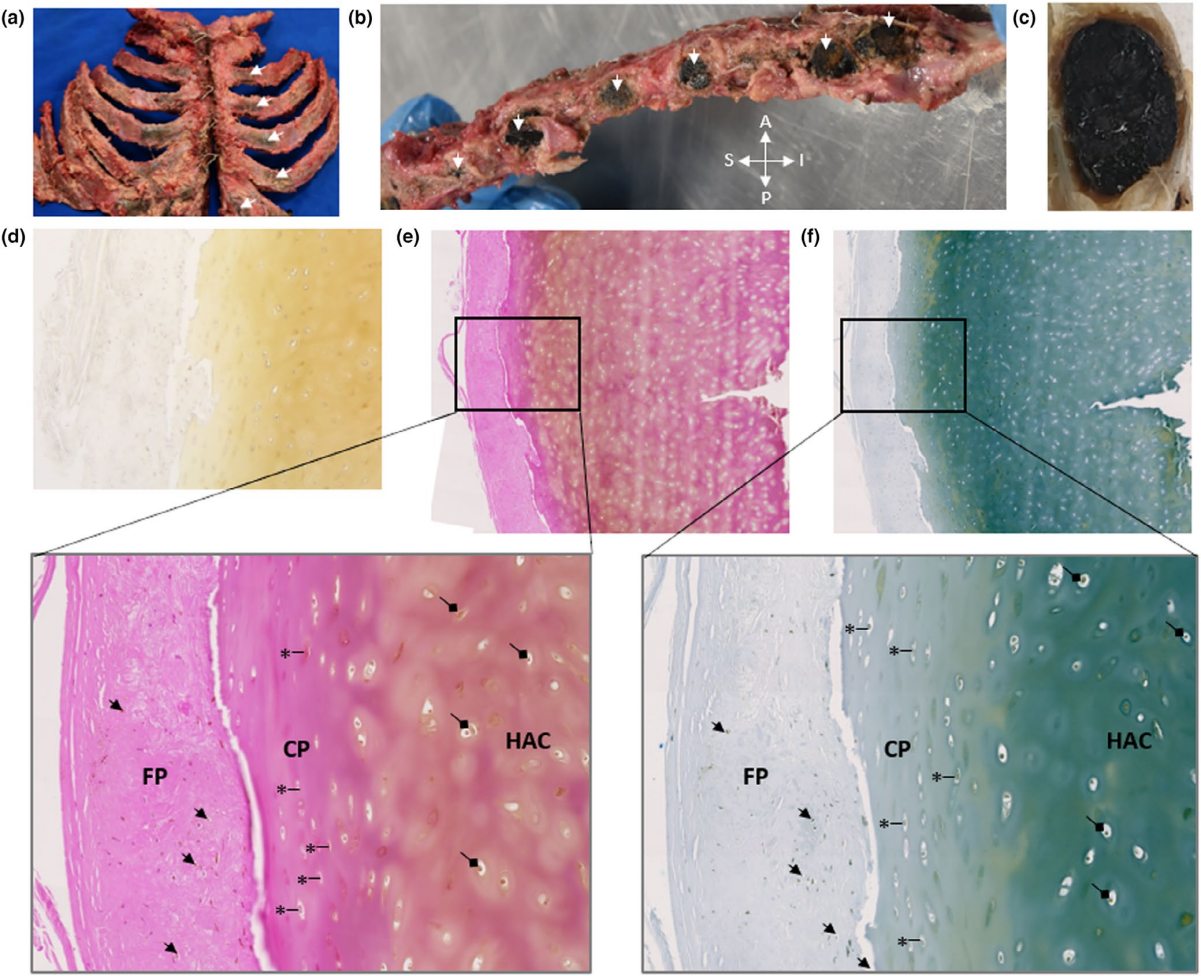
Intense ochronotic pigmentation of costal cartilage. An anterior view of the sternum and associated costal cartilage and ribs is shown in (a), with intense black pigmentation of costal cartilage indicated by arrows on the left side, with pigment also present on the right side. A lateral view of the sternum with the ribs removed at the sternocostal junctions is shown in (b), with arrows indicating black pigmented cartilage (A = anterior, P = posterior, S = superior, I = inferior). The costal cartilage of left second rib was transected and is shown in (c) with intense black pigmentation observed throughout the cartilage. Histological sectioning of the left second costal cartilage in (c) is shown in (d) with no staining where pigment appears a yellow‐ochre colour, in (e) with H&E staining and (f) with Schmorl's staining, with the insets shown at greater magnification below. Intense pigmentation can be observed throughout the hyaline articular cartilage (HAC) of the costal cartilage, within chondrocytes (indicated by diamond arrows) and spread through the entire matrix. Surrounding the HAC is a thick perichondrium, divided into two layers; an outer fibrous perichondrium (FP) where pigmentation is mostly associated with fibroblast cells (examples indicated by arrows) and only a small amount in the matrix, and an inner chondrogenic perichondrium (CP), where pigmentation is both within chondroblasts (examples indicated by an asterisk) and throughout the matrix.

All costal cartilage observed was intensely pigmented and black in colour, both at the costal margin and sternocostal junctions (Figure 4a,b), and was very hard and brittle. An unstained section of the black cartilage of the left second sternocostal junction shown in Figure 4c, histologically shows golden yellow or ochre coloured pigment, spread throughout the entire cartilage matrix (Figure 4d). H&E and Schmorl's staining of the same area confirmed that chondrocytes and the entire HAC matrix were pigmented (Figure 4e,f). A clear delineation between the outer and inner layers of the thick perichondrium present around costal hyaline cartilage was observed (Figure 4e,f). The inner chondrogenic layer showed pigmented chondroblasts and pigmented extracellular matrix. The outer fibrous perichondrium showed pigmented fibroblasts and little pigmentation within the extracellular matrix.

### 3.5 Elastic cartilage

**FIGURE 5:**
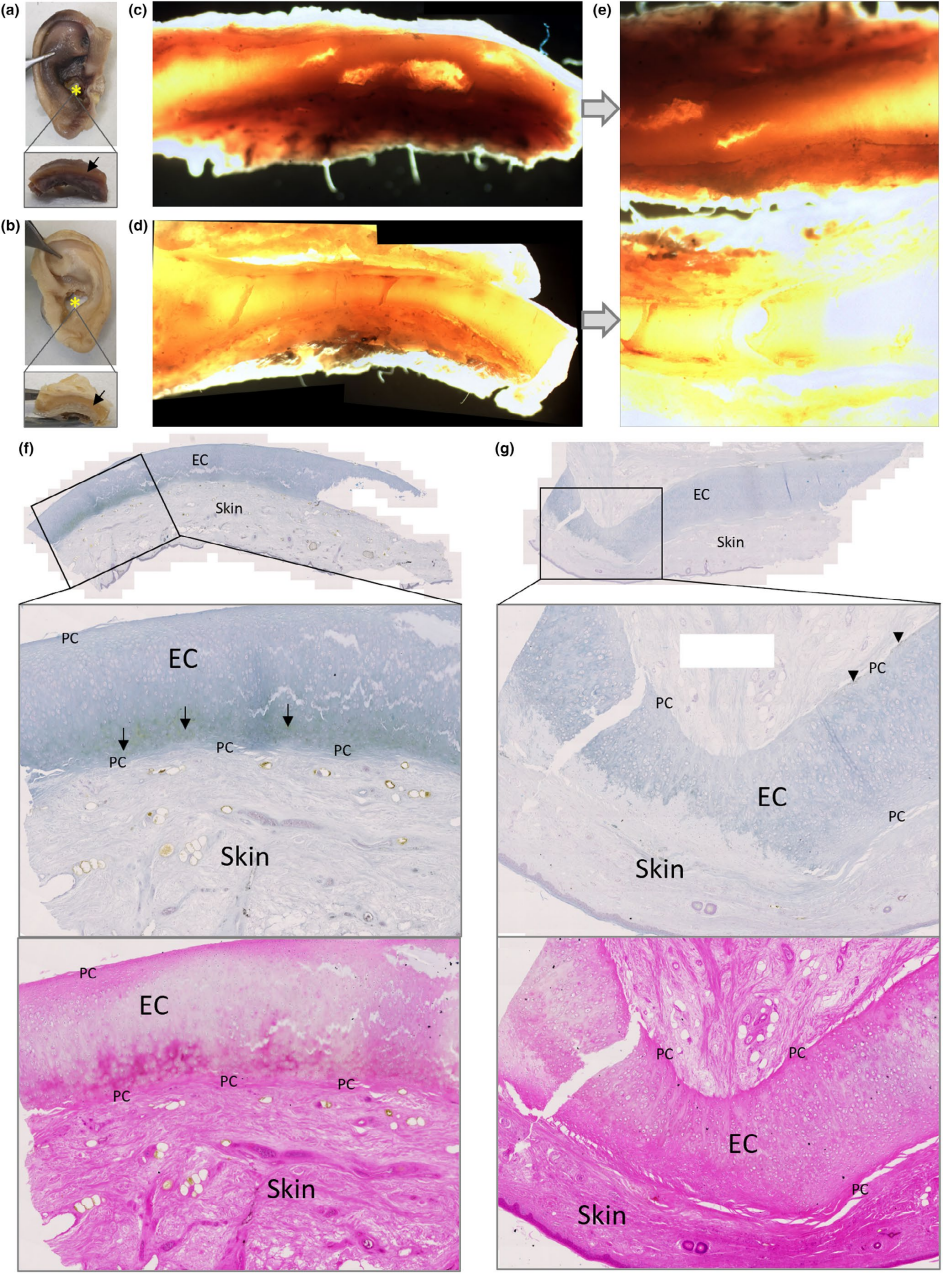
Ochronotic pigmentation of elastic cartilage from the ear. The right and left intact auricles are shown in (a) and (b) respectively (post‐fixation). The asterisk indicates where a circular biopsy approximately 1 cm in size was removed from the conchal bowl of each auricle, and then bisected, with the cross section shown in the inset and the arrow indicating the elastic cartilage. A thin slice of each biopsy was examined with a dissecting microscope using dark field illumination and photographed; right shown in (c) and left shown in (d), with the right appearing much darker suggesting more pigmentation. A direct comparison of the right (top) and left (bottom) biopsies is shown in (e). Histological sections of the right and left conchal bowl biopsies, stained with Schmorl's stain, are shown in (f) and (g), respectively, with the inset shown at greater magnification below with a corresponding H&E stained image of the same area. Arrows in (f) demonstrate the most pigmented area of the elastic cartilage (EC), adjacent to perichondrium (PC). Arrow heads in (g) indicate mild pigmentation of perichondrium.

**FIGURE 6:**
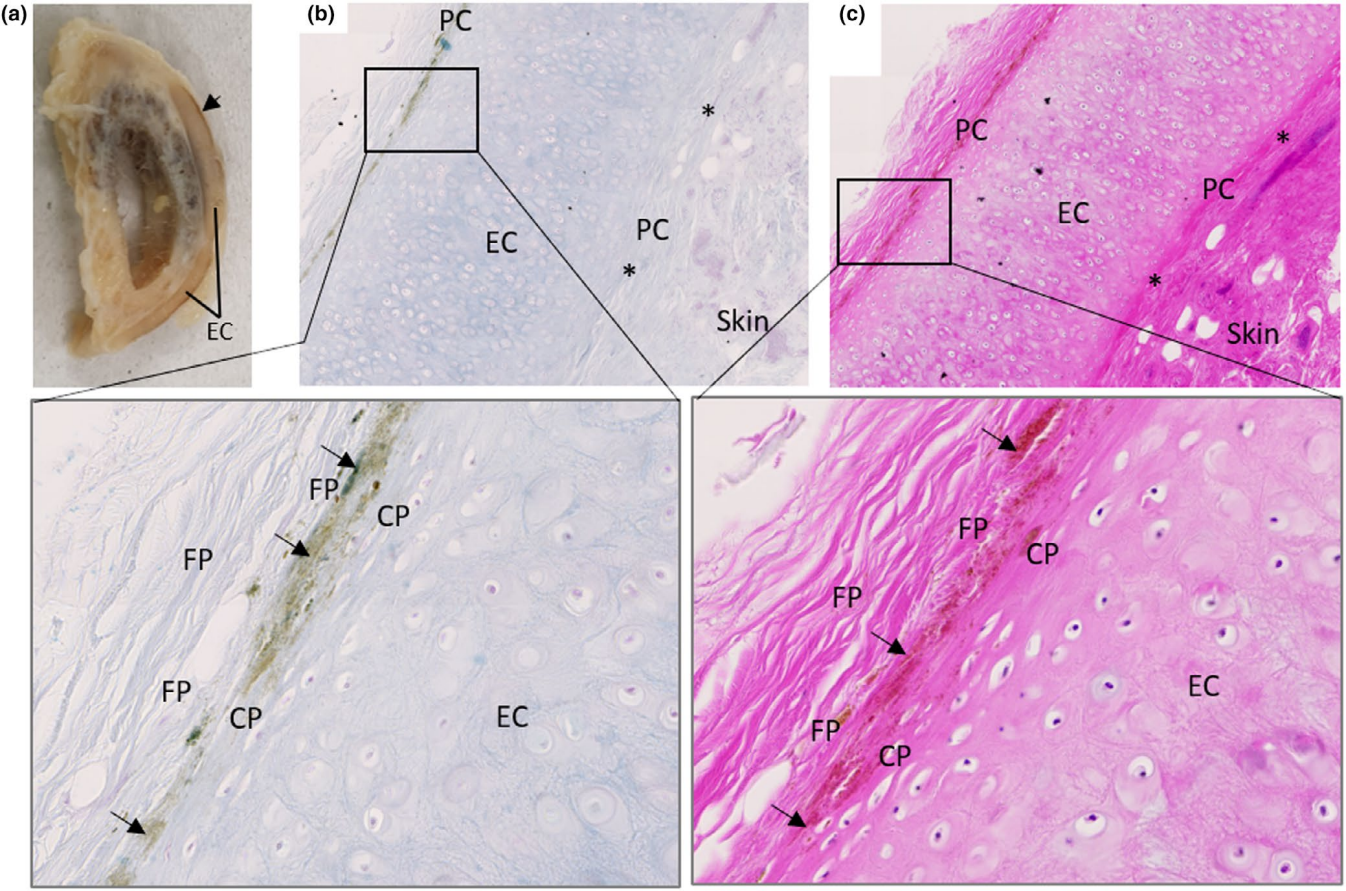
Ochronotic pigmentation of elastic cartilage perichondrium. A sagittal section through the right external acoustic meatus is shown in (a), with the arrow highlighting an area of pigmentation observed macroscopically at the periphery of the elastic cartilage (EC). Schmorl's staining of the external acoustic meatus elastic cartilage (EC) is shown in (b) with a corresponding area stained with H&E in (c). Pigmentation only appears to be associated with the perichondrium, situated mostly within the outer fibrous perichondrium (FP) adjacent to the boundary with the inner chondrogenic perichondrium (CP), although some appear to be located within the chondrogenic perichondrium. The asterisk indicates areas of perichondrium that are not pigmented.

Grossly, the right ear overall was much darker than the left ear, with the cartilage also appearing darker in colour when a circular biopsy from the conchal bowl of each ear was bisected (Figure 5a,b). A thin slice from each conchal biopsy was examined via dark field illumination using a dissection microscope (Figure 5c,d), with a direct comparison shown in Figure 5e. The cartilage pigmentation was patchy and unevenly distributed, and more intense in the right biopsy, which was confirmed with Schmorl's staining (Figure 5f,g), with an area of more intense pigmentation highlighted by arrows in Figure 5f. Small areas of the perichondrium were pigmented in both biopsies, see the arrow heads in Figure 5g. The skin adjacent to the cartilage appeared unpigmented in both biopsies. Grossly, pigmentation was only observed at the periphery of the elastic cartilage of the external acoustic meatus in the perichondrium (Figure 6a), also shown histologically in Figure 6b,c, with the elastic cartilage itself appearing normal. Note that some of the perichondrium remained unpigmented. Histologically, no pigmentation was identified within the external acoustic meatus cartilage chondrocytes or extracellular matrix.

### 3.6 Respiratory cartilage

**FIGURE 7:**
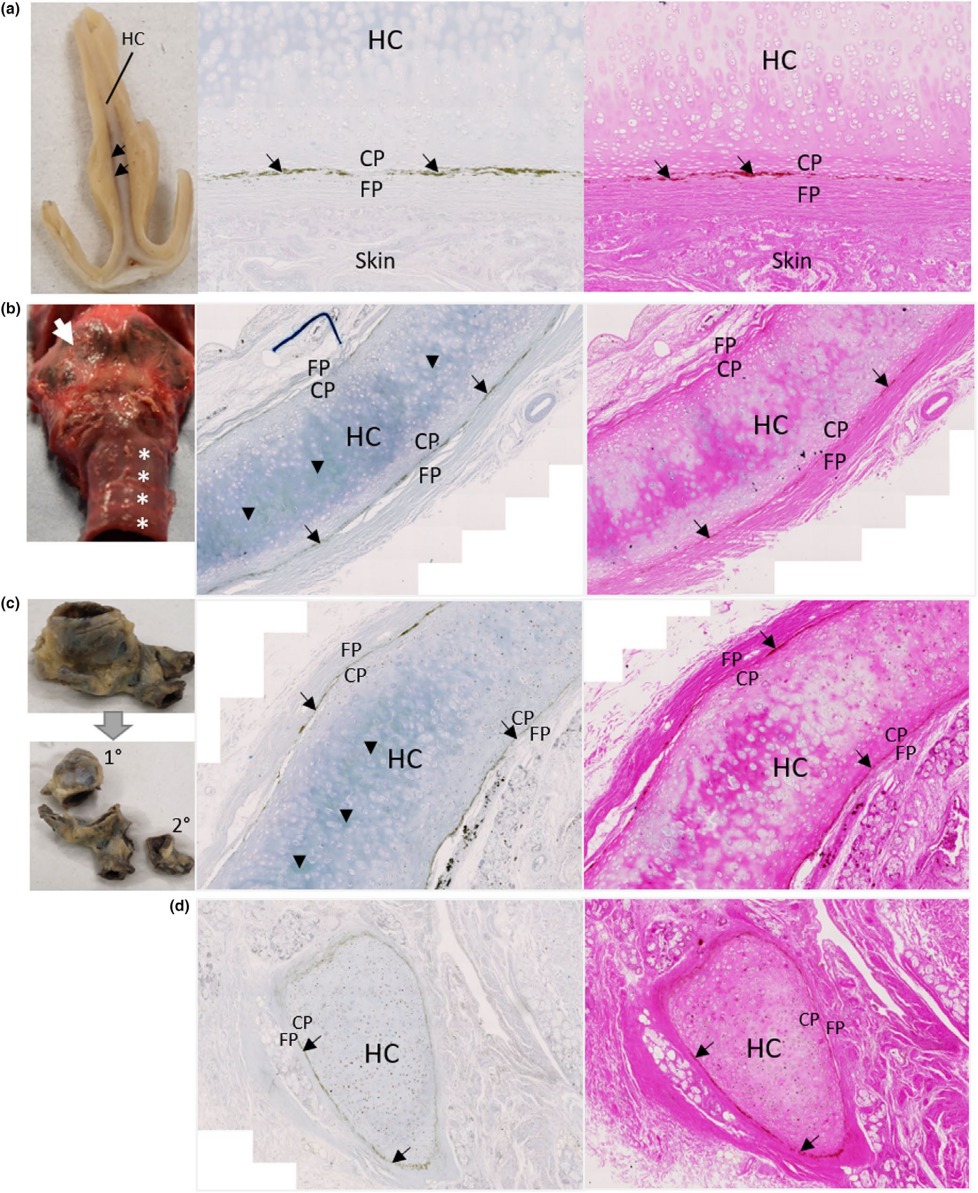
Ochronotic pigmentation of hyaline respiratory cartilage and perichondrium. Gross images of hyaline cartilage from the respiratory tract are shown on the left of each row, with corresponding Schmorl's (middle) and H&E (right) staining. A coronal section through the nasal septum (post‐fixation) is shown in (a) with the location of hyaline cartilage (HC) shown and arrows indicating pigmentation at the periphery of the HC. Histologically, no pigmentation was observed within the HC, but was observed within the perichondrium, situated at the boundary between the fibrous perichondrium (FP) and the chondrogenic perichondrium (CP). The larynx and proximal trachea (pre‐fixation) are shown in (b), with the arrow indicating the thyroid cartilage and the asterix's indicating the most superior tracheal rings, all of which appear black with pigmentation. The histology in (b) shows a tracheal ring from the superior mediastinum, with pigmentation observed within the hyaline cartilage (HC) indicated by arrow heads, and arrows indicating pigmentation of the perichondrium located between the FP and CP. A segment of the left bronchial tree, with primary (1°) and secondary (2°) bronchi isolated, is shown in (c), although the cartilage cannot be observed grossly from this view. Pigmentation was observed within the primary bronchial cartilage as indicated by arrows heads in row (c) via Schmorl's staining, but not within secondary bronchial cartilage in row (d). Pigmentation was observed in the perichondrium between the FP and CP in both primary and secondary bronchial cartilage.

Hyaline cartilage of the respiratory tract was examined. Dark pigmentation at the periphery of the nasal septum cartilage was observed grossly, with histological analysis showing no pigmentation associated with the chondrocytes or the cartilage matrix (Figure 7a). Grossly, the thyroid cartilage of the larynx and adjacent upper tracheal rings were intensely pigmented and were black in colour (Figure 7b). Histologically, cartilage pigmentation of a superior mediastinum tracheal ring was identified (Figure 7b). Pigmentation was observed in cartilage from the primary bronchus of the left bronchial tree, but was not identified in secondary bronchus cartilage (Figure 7c,d). Perichondrium pigmentation was observed in all of the respiratory cartilages examined histologically (nasal septum, tracheal ring, bronchial cartilage), situated between the inner chondrogenic and outer fibrous layers (Figure 7a–d).

### 3.7 Upper limb joints

**FIGURE 8:**
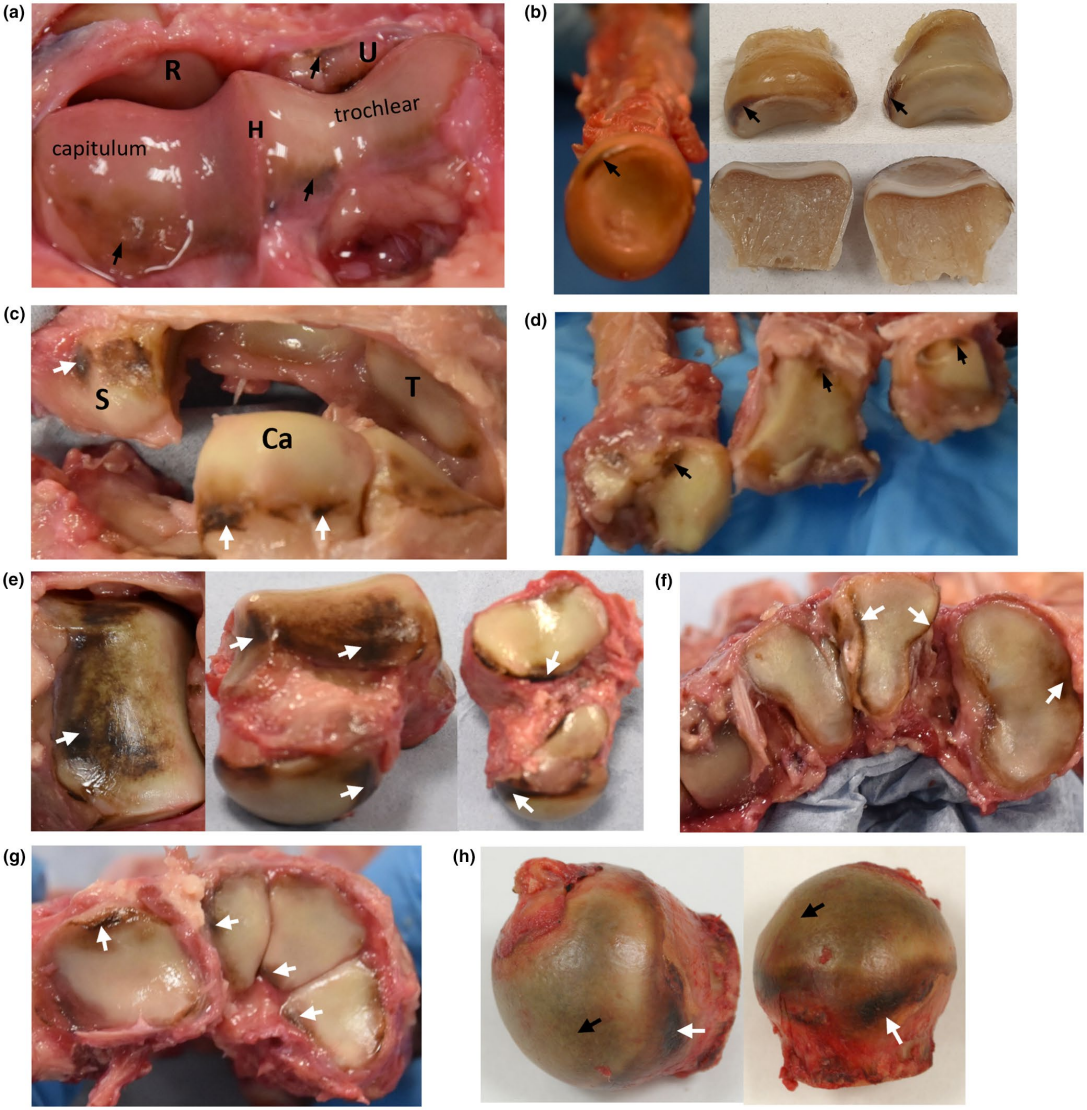
Ochronotic pigmentation of upper and lower limb articular cartilage and perichondrium. A superior view of the distal end of the left humerus (H) is shown in (a), with pigmentation indicated by arrows on the articular cartilage of the humerus. The proximal ends of the radius (R) and ulna (U) are visible. A superior view of the radial head is shown in (b) (left), which has been bisected (right), with pigmentation of articular cartilage indicated by arrows. The scaphoid (S), capitate (Ca) and triquetral (T) carpal bones are shown in (c), with arrows indicating dark pigmentation that is located at the periphery of the articular surface, adjacent to the bone. The proximal ends of the 2nd to 4th metacarpal bones, from left to right, are shown in (d), with pigmentation present at the edge of the articular surfaces, indicated by arrows. From left to right, (e) superior, anterior and inferior views of the talus with arrows indicating areas of pigmentation. The proximal ends of the first to fourth metatarsals (from right to left) are shown in (f), with arrows indicating pigmentation. The proximal ends of the cuboid, medial cuneiform, intermediate cuneiform and lateral cuneiform tarsal bones (from left to right) are shown in (g). The femoral head of the hip joint is shown in (h), with black arrows highlighting pigmentation of the main articular surface, and the white arrows indicating pigmentation of perichondrium. All gross images were taken pre‐fixation, except the bisected radial head images taken post‐fixation in (b).

The left upper limb was examined grossly, with the shoulder joint excluded from dissection due to it being artificial. Pigmentation of the articular cartilage of the distal humerus, radial head and proximal ulna was observed as golden brown to dark brown pigment (Figure 8a,b). Overall, pigmentation was confined mostly to the edges of the articular surfaces of upper limb joints, see Figure 8a,c,d, although in the radial head, dark pigmentation was observed on the main articular surface, see arrows in Figure 8b. Most of the articular cartilage of the right capitate (left was unavailable for histological examination) grossly appeared unpigmented, but when examined histologically pigmentation of chondrons was observed in the CAC with both intracellular, pericellular and territorial matrix pigmentation (Supplementary Figure S3). Histological examination of the distal fifth interphalangeal joint revealed pigmented chondrons within the articular cartilage (data not shown).

**FIGURE 9:**
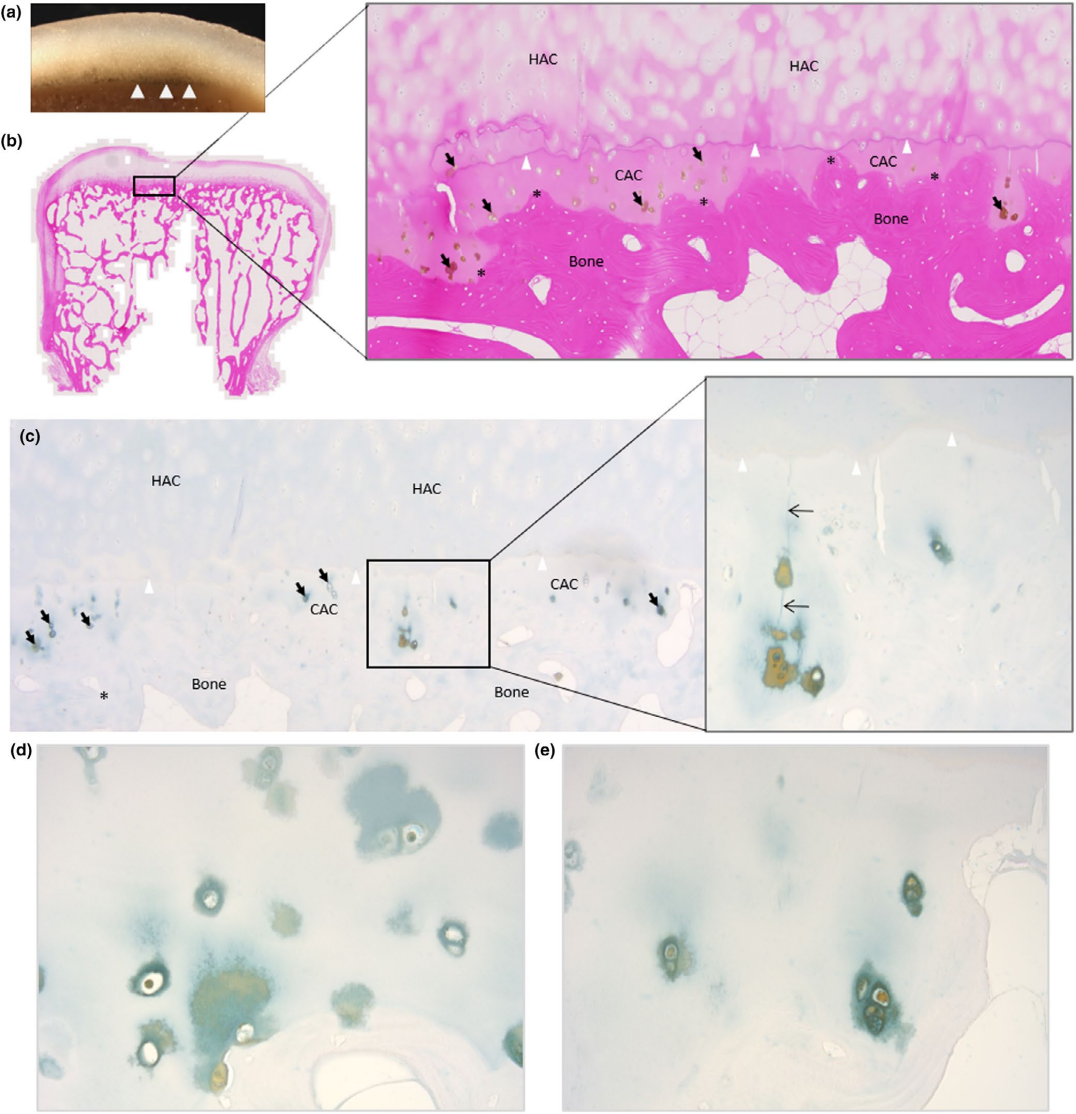
Ochronotic pigmentation of calcified articular cartilage. A cross section through the articular surface of the left radial head (post‐fixation) is shown in (a), with the white arrow heads indicating pigmentation of the deep calcified articular cartilage adjacent to the subchondral bone that appears unpigmented. H&E staining of the radial head in is shown in (b) and Schmorl's staining in (c). Pigmented chondrons, indicated by arrows, in (b) and (c) are observed in the calcified articular cartilage (CAC), deep to the tidemark indicated by white arrow heads, and not in the hyaline articular cartilage (HAC). Schmorl's staining of the articular cartilage is shown in (c). Asterisks indicate the position of the cement line separating the CAC from the underlying subchondral bone. The inset in (c), along with (d) and (e), show higher magnification images of pigmented chondrons within the CAC. The arrows in the inset of (c) show a pigmented line connecting adjacent pigmented chondrons and the superficial tidemark.

When bisected and photographed, macroscopic pigmentation could be observed deep within the radial head articular cartilage, immediately adjacent to the unpigmented subchondral bone (Figure 9a). Figure 9b–e shows pigmentation associated with chondrocytes in the CAC, and not within the HAC superficial to the tidemark. Pigmentation is more advanced in some chondrons than others, with only pericellular pigmentation observed in some chondrons compared to pigment spreading into the territorial and inter‐territorial matrix of other chondrons. Pigmented chondrons were observed adjacent to the cement line, with the most heavily pigmented chondrons in Figure 9b appearing to be in the deepest areas of the CAC, furthest from the tidemark; this was also observed in the capitate (Supplementary Figure S3a). The tidemark was also duplicated, and in some regions, there were three tidemarks (not shown), with a few lightly pigmented chondrons observed between the tidemarks (Figure 9, Supplementary Figure S3). In Figure 9c, heavily pigmented chondrons were observed (see the inset), with the pigment remaining unstained with Schmorl's stain and appearing dark gold‐brown in colour. A noteworthy feature was also identified, with a line of pigmentation appearing to bridge vertically between two pigmented chondrons and from the most superficial chondron to the tidemark, see the black arrows in inset of Figure 9c.

Perichondrium is associated with the periphery of the articular surface where cartilage is adjacent to bone, and dark pigmentation of perichondrium was observed. For example, the arrows in Figure 8c demonstrate dark, superficial pigmentation of the perichondrium on the capitate, at the junction of the cartilage and bone shaft. The radial head was cut into two segments and dark pigment was clearly observed on the superficial surface of the cartilage (see arrows Supplementary Figure S5a) at the edge of the articular surface towards the bone shaft, and not within the deeper cartilage which appears white on its cut surface. The periosteum is visible more distally, which appears whiter. The radial head was examined histologically at the periphery of the articular surface (dashed box area Supplementary Figure S5a), where pigmentation was observed at the superficial surface of the cartilage, indicated by arrows, which became fainter towards the main articular surface further away from the bone shaft (Supplementary Figure S5b). The pigment is located within the inner chondrogenic portion of the perichondrium, with the outer fibrous perichondrium appearing unpigmented. At the junction of the perichondrium and periosteum (Supplementary Figure S5c), a diffuse, granular‐like pigmentation was observed within the perichondrium and adjacent hyaline articular cartilage. More distally, pigmentation within the periosteum was observed (Supplementary Figure S5d).

### 3.8 Lower limb joints

The left lower limb was dissected and examined, excluding the hip and knee joints which were artificial (in both lower limbs). The individual had donated the right femoral head after an arthroplasty surgery approximately 3 years prior to death, therefore this was available for gross examination. The right femoral head had intact cartilage that appeared to be pigmented across the centre of the articular surface (black arrows, Figure 8h) with very dark pigmentation of the perichondrium (white arrows) at the periphery of the articular surface adjacent to the bony neck of the femur. Only the ankle joint and joints within the foot were available to examine in the left lower limb; of these, the talus of the ankle joint was the most pigmented articular joint surface observed, with black to brown pigmentation across the talocrural joint surface (Figure 8e). All other talar articular surfaces were pigmented, confined to the periphery of the articular surfaces. Pigmentation was observed on all other tarsal bone articular surfaces and the heads of the metatarsals, varying from light brown to dark brown/black, particularly at the edges (Figure 8f,g). An intensely pigmented inter‐tarsal ligament insertion was observed on the lateral cuneiform which was black in colour (white arrow, Supplementary Figure S4a). Pigmentation of chondrocytes within the CAC was observed histologically in the lateral cuneiform (not shown). Pigmentation was present on the periphery of the lateral cuneiform's articular surface (see dashed box area of Supplementary Figure S4a) and was superficial; histological examination of the lateral cuneiform in this region (Supplementary Figure S4b‐c) showed more intense pigmentation at the periphery of the articular surface (left‐hand side of the image).

### 3.9 Bone

Bone associated with the joints examined above grossly appeared normal. The periosteum was only investigated histologically in the radius, as mentioned above. Trabecular bone of the radial head, capitate and lateral cuneiform examined histologically showed some pigmentation of osteocytes (Supplementary Figure S6c,g,f), observed only in a few regions on each of the sections examined, with the majority of osteocytes appearing unpigmented. In the ear ossicles (Figure 4), pigmented osteocytes were much more abundant than those observed in the limbs. Within the malleus (Figure 4a), pigmented osteocytes were more frequent in the handle and neck of the malleus. The pigmented osteocytes in the incus and stapes appeared to be distributed throughout the bone matrix (Figure 4b,c). Pigmentation of large, round cells assumed to be chondrocytes was observed in areas of trabecular bone matrix of the lateral cuneiform and radial head that did not stain strongly with eosin and did not have a lamellar structure (Supplementary Figure S6b,d), suggesting that these regions could be remnants of calcified cartilage or old woven bone that did not get replaced with lamellar bone. Schmorl's staining confirmed pigmentation of the osteocytes and chondrocytes.

### 3.10 Other tissues and viscera

**FIGURE 10:**
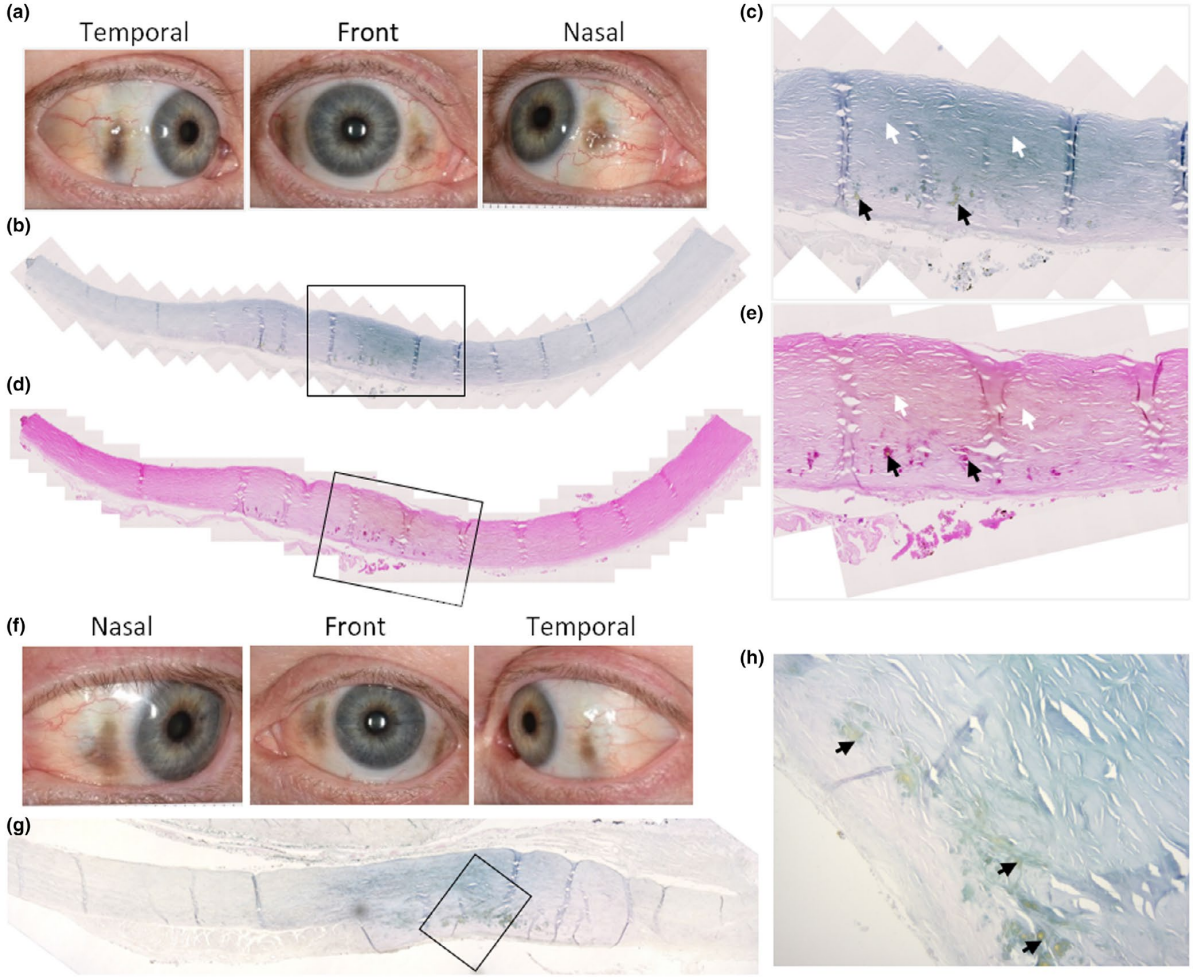
Ochronotic pigmentation of the sclera. Photographs of the right eye antemortem are shown in (a), showing ochronotic pigmentation in the nasal and temporal regions of the sclera. Histological sections of the right temporal sclera showing ochronotic pigmentation, stained with Schmorl's stain in (b) and H&E in (d) are shown. The insets in (b) and (d) are shown in (c) and (e), respectively, with white arrows indicating diffusely pigmented areas, and black arrows indicating focal pigmentation. Photographs of the left eye antemortem are shown in (f), showing ochronotic pigmentation in the nasal and temporal regions of the sclera. A histological section of pigmented sclera from the left eye stained with Schmorl's stain shown in (g), with a higher magnification image shown in (h) where diffuse pigmentation is observed throughout the sclera, and focal aggregates of pigment are indicated by arrows. Antemortem photographs of the eyes were taken 9 months prior to death.

During life, pigmentation of the sclera in the temporal and nasal region was observed in both eyes and photographed, see Figure 10a,f. Histological sectioning of the right temporal sclera shows the presence of ochronotic pigment, with Schmorl's staining (Figure 10a,c) showing a region of intense staining, that corresponds to an area of yellow coloured pigment in the H&E stained section (Figure 10d,e). Figure 10g shows a region of pigmented sclera (left eye) stained with Schmorl's stain, with the higher magnification image showing both diffuse and focal pigmentation, with the focal accumulations appearing more intense and yellow/brown in colour (Figure 10h).

Overall, the viscera appeared normal and healthy. Unless stated otherwise, the following viscera were examined grossly and histologically via H&E and Schmorl's staining. The oesophagus, stomach, duodenum, jejunum, ileum, large intestine, liver, gallbladder, pancreas, spleen, kidney, bladder, ovary, skeletal muscle (gastrocnemius, adjacent to Achilles tendon), salivary glands (parotid, sublingual, submandibular), lacrimal gland and lymph nodes (carotid sheath and tracheal) appeared normal and unpigmented (Supplementary Figures S7–13). The stomach omenta and appendix appeared normal grossly but were not examined histologically. The lung parenchyma appeared unpigmented (Supplementary Figure S11a), with the cartilage of the bronchi and trachea described separately above. The uterus was only examined grossly and appeared unpigmented, with the only abnormal feature observed being a large fibrous cyst (fibroid) on the anterior part that upon transection appeared non‐pigmented. The brain was removed from the skull and not examined further, but grossly appeared unpigmented.

## 4 DISCUSSION

We have carried out an extensive anatomical and histological examination of tissues from an AKU individual, aged 60 years. The detrimental effect of pigmentation had already begun to take its toll on this individual; six major joint replacements had been carried out (both knees, hips and shoulders) and aortic valve function had declined requiring aortic valve replacement surgery, during which this individual passed away. Determining how, why and when, HGA‐derived pigment targets specific osteochondral tissues, is essential to understanding the processes that lead to accelerated joint destruction in AKU. We highlight the asynchronous nature of ochronotic pigmentation of connective tissues across the body, with some tissues exhibiting severe pigmentation whilst others were unaffected. Within articular cartilage, we confirm that focal pigmentation of calcified articular cartilage chondrons and their surrounding matrix occurs before pigmentation of the superficial non‐calcified cartilage. Determining why pigmentation occurs in this order within joints will be key to understanding the mechanism of accelerated joint destruction in AKU, which may provide insight into other common degenerative disorders such as OA. Intense pigmentation of non‐articular hyaline cartilage perichondrium was observed, with our study suggesting that perichondrium of these cartilages is affected prior to the cartilage itself. We show novel histological images of the ear ossicles, tympanic membrane, pubic symphysis and sclera from an AKU individual (all pigmented), with a surprising lack of pigmentation in the temporomandibular joint and numerous pigmented osteocytes within the ear ossicles.

### 4.1 Articular cartilage

The articular cartilage of large weight‐bearing joints is the most affected tissue in AKU; with OA predominately affecting the spine, knees, hips and shoulders (Ranganath et al., 2021). The joints examined here that appeared to show the early stages of ochronosis confirmed that the pigmentation of articular cartilage begins in the CAC associated with chondrons and the immediately adjacent territorial matrix, observed previously in human tissues (Taylor et al., 2011) and in AKU mice (Hughes et al., 2021; Preston et al., 2014). We also provide further evidence that the larger, more load‐bearing joints are affected more severely by ochronosis; this individual had already undergone six major joint arthroplasties suggesting they were both ochronotic and osteoarthritic, and of the joints examined the femoral head and talus of the ankle were the most pigmented, followed by smaller joints such as the elbow and those of the hands and feet. This demonstrates the asynchronous nature of pigmentation across the body; with greater load speculated to be the causative factor of the heterogeneity (Helliwell et al., 2008; Ranganath et al., 2021; Taylor et al., 2011).

The tidemark, or current mineralising front as it is also known, is the interface between the HAC and CAC. Tidemark duplication was observed histologically in several articular cartilage samples, with pigmented chondrons located between the two tidemarks, with three observed in some cases. Tidemark duplication or advancement is often observed in OA (Oettmeier et al., 1989), with a dual tidemark associated with a higher OARSI grade in human joints (Palmer et al., 2014). Tidemark multiplication, however, can also be considered a normal feature of articular cartilage (Boyde, 2021; Lane & Bullough, 1980; Oegema et al., 1997). Palmer et al. (Palmer et al., 2014) suggest that tidemark advancement occurs at an early stage of OA and may perpetuate further osteoarthritic changes in the HAC in a ‘bottom‐up’ mechanism. We know that AKU joints become osteoarthritic due to pigment deposition in the CAC before the HAC also in a ‘bottom‐up’ manner, therefore it is plausible that the tidemark multiplication observed here is pathological. Interestingly, pigmented chondrons were observed between two tidemarks within CAC; whether these chondrons become pigmented after tidemark advancement/matrix calcification or before when they were still HAC chondrocytes remains unknown. We speculate that it is likely after tidemark advancement, as no pigmented chondrons were observed in the adjacent HAC.

It is possible that the low oxygen tension (Brighton et al., 1971; Zhou et al., 2004) and acidic pH, reported to be as low as 6.6–7.2 (Gray et al., 1988; High et al., 2019; Razaq et al., 2003; Urban et al., 1993), of cartilage may promote pigmentation, however, other factors must be involved to explain why CAC pigments before the neighbouring HAC. Although CAC has historically been disregarded in joint/cartilage research due to difficulty evaluating it, a recent review by Evans and Pitsillides (Evans & Pitsillides, 2022) highlights the importance of this osteochondral interface tissue. The importance of CAC towards joint pathologies such as OA has begun to be uncovered, with examination of osteoarthritic mice also showing early pathological changes in CAC that precede OA (Herbst et al., 2023; Madi et al., 2020; Staines et al., 2016). Here, we observed that the most intensely pigmented CAC chondrons were situated in the deepest layers of the CAC where the undulating cement line is deeper, and therefore a further distance from the tidemark. Whether this is significant towards the pathological mechanism of ochronosis remains unknown but may warrant further investigation with examination of samples from different AKU donors.

### 4.2 Non‐articular cartilage

In addition to CAC, we present other skeletal connective tissues and cells that pigment, such as perichondrium, non‐articular hyaline cartilage, fibrocartilage and bone osteocytes. Histologically, intense pigmentation of non‐articular hyaline cartilage appeared widespread and diffuse rather than focal, similar to the previously described blanket pigmentation of HAC (Taylor et al., 2011), suggesting that they are similar. Once HAC begins, it is reported to progress and spread rapidly throughout the matrix, faster than the underlying focal CAC (Taylor et al., 2011). The black appearance of the non‐articular hyaline cartilage in this examination (i.e. trachea, costal) would support this, as it was more intense and widespread than the focal pigment observed in joint CAC. Why the non‐calcified hyaline cartilage of joints is initially resistant to pigmentation is not known, and we suggest that changes to the matrix may occur, allowing for this rapid pigmentation.

### 4.3 Perichondrium

Perichondrium is the lining surrounding non‐articular hyaline and elastic cartilage and is composed of an outer fibrous layer with an inner chondrogenic or cellular layer (Ross & Pawlina, 2015). Although articular cartilage is devoid of perichondrium on the main articular surface, it is located at the periphery of articular cartilage, adjacent to the bone, where it blends with periosteum. Pigmentation of perichondrium related to joints has been reported by Lichtenstein and Kaplan (Lichtenstein & Kaplan, 1953). Pigmentation of the perichondrium of respiratory hyaline cartilage and costal cartilage has been described but not discussed, with the few available images showing it to be associated with the inner perichondrium layer as we have described, spreading into the hyaline cartilage matrix (Gaines, 1989; Helliwell et al., 2008; Lichtenstein & Kaplan, 1953; Nishimori et al., 1970). Comparable to a description by Gaines (Gaines, 1989), we showed more prominent pigmentation in the perichondrium of multiple cartilages than in the cartilage itself, and show cartilages with pigmentation present only within the perichondrium. This indicates that pigment deposition occurs in the perichondrium before the cartilage matrix in the non‐articular cartilages that we examined. One exception was the costal cartilage, where more intense pigmentation in the hyaline cartilage was observed compared to the perichondrium, therefore we hypothesise that although perichondrium pigmentation precedes cartilage pigmentation in these tissues, it proceeds more rapidly in the hyaline cartilage.

### 4.4 Tympanic membrane and ear ossicles

Tympanic membrane discolouration in AKU is well documented in the literature (Al‐Shagahin et al., 2019; Ozer Ozturk et al., 2022; Pau, 1984; Sagit et al., 2011; Sanji et al., 2011; Steven et al., 2015). We demonstrate novel pigmentation of the ear ossicles, located within their osteocytes (bone is discussed below), and within the CAC at the inter‐ossicular joints, and of the tympanic membrane and its connection to the malleus. The tympanic membrane and synovial joints of the ear ossicles would be under constant strain from sound vibrations, therefore although they are small, it is not surprising to find pigmentation here. Previous biomechanical research has shown that AKU articular cartilage is stiffer than non‐AKU and OA cartilage (Taylor et al., 2011). Stiffening of the tympanic membrane and ear ossicle chain could therefore be occurring in AKU patients, which could have an impact on hearing.

Mild to moderate hearing loss in AKU has been shown in the literature, with an incidence of 35% (*n* = 20) and 44% (*n* = 16) in two studies, at an average age of 63 and 54 years respectively (Al‐Shagahin et al., 2019; Steven et al., 2015). We would propose that pigmentation of the tympanic membrane and synovial ear ossicle joints would cause conductive hearing loss affecting sound conduction through the outer and middle ear cavities, with abnormal tympanograms. Surprisingly, the majority of the hearing loss was sensorineural in these studies, with just a few cases reporting mixed hearing loss (combination of conductive and sensorineural) (Ozer Ozturk et al., 2022; Pau, 1984; Sagit et al., 2011), all with normal tympanograms, suggesting that the inner ear/central nervous system may in fact be involved. Cochlear function could be affected by HGA and/or pigmentation, but this has never been assessed. The absence of acoustic reflexes in the two cases reporting mixed frequency hearing loss with normal tympanograms (Ozer Ozturk et al., 2022; Sagit et al., 2011) could point to stiffness/abnormal function of the ear ossicle chain that prevents the acoustic reflex form acting on the tympanic membrane, or perhaps even stiffness of the tensor tympani/stapedius tendons themselves. The mechanism in which hearing is impaired in AKU remains to be established.

### 4.5 Bone

We did not observe macroscopic or microscopic pigmentation of the bone matrix, which is rich in type I collagen, agreeing with previous macroscopic observations (Helliwell et al., 2008; Lichtenstein & Kaplan, 1953), with the assumption that the mineralisation of the matrix is protective. Osteocyte pigmentation was observed in trabecular bone of appendicular joints and the ear ossicles, also observed by Taylor et al. (Taylor et al., 2011). Osteocyte pigmentation in the trabecular bone from limbs was scarce in comparison to the numerous pigmented osteocytes observed in the ear ossicles. The unique function and composition of the ear ossicles may explain why they are more susceptible to pigmentation.

The ear ossicles reach their final shape and size by birth which is unusual for most bones. The ossicles are composed exclusively of compact bone, with no trabecular bone, and are formed of a mixture of lamellar and woven bone (Duboeuf et al., 2015; Hassmann & Chodynicki, 1978). Two human ear ossicle studies reported low levels of bone remodelling (Chen et al., 2008; Duboeuf et al., 2015). Two other studies showed that empty osteocyte lacunae increase dramatically from birth via apoptosis in human ear ossicles, in addition to the number of degenerating osteocytes, with no apoptosis observed in older age (i.e. 60 years), with the hypothesis that osteocyte apoptosis is a programmed phenomenon whereby cell death inhibits bone remodelling, ensuring that the ear ossicles acquire the structural stability they need for sound transmission (Marotti et al., 1998; Rolvien et al., 2018). In AKU, lack of bone turnover would result in older osteocytes, with longer HGA exposure, and more opportunity to become pigmented, compared to osteocytes of newly formed bone. Additionally, it has been shown that osteocyte lacunae of ear ossicles are mineralised/micropetrotic, with most of these changes occurring within the first year of life (Rolvien et al., 2018), occurring after osteocyte cell death (Milovanovic & Busse, 2023). This suggests that many, if not most of the pigmented osteocytes observed here within the ear ossicles might have been dead. Whether they become pigmented before or after death cannot be deduced via our observations, as we did not look at viability, and the tissue was decalcified for examination.

At a few sites within the appendicular trabecular bone examined, pigmented osteocytes surrounded by fully mineralised lamellar bone were observed. These osteocytes tended to be grouped together, suggesting an area of older bone with longer lived osteocytes that had time to pigment. Additionally, there were areas within the trabecular bone network where pigmentation appeared to be associated with rounder, chondrocytic‐looking cells surrounded by a non‐mineralised matrix that stained weakly with eosin and lacked a lamellar structure. This matrix appears to be remnant calcified cartilage that has not been replaced by lamellar bone and is susceptible to pigmentation.

### 4.6 Fibrocartilage

Fibrocartilage is composed of dense fibrous tissue with chondrocytes and fibroblasts that are surrounded by a matrix that contains type I collagen in addition to type II collagen, designed to resist compression, shear force and tension. Previously, pigmentation has been described in intervertebral discs (Galdston et al., 1952; Helliwell et al., 2008), the pubic symphysis (Helliwell et al., 2008; Lichtenstein & Kaplan, 1953) and knee joint menisci (Nag et al., 2013; Rajani et al., 2021; Xu et al., 2015). Pigmentation of the pubic symphysis disc was observed here, and appeared patchy, with focal deposits associated with cells, agreeing with previous reports. We were therefore surprised to find no pigmentation within the fibrocartilage TMJ disc. Although its biomechanics are complex, the TMJ is considered a loaded joint and TMJ fibrocartilage disc has an extracellular matrix composition of type I collagen and elastin, with only trace amounts of type II collagen and other collagens (Detamore & Athanasiou, 2003; Landesberg et al., 1996; Runci Anastasi et al., 2021), suggesting it is subjected to high tensile strain and deformation. We cannot say with certainty that pigmentation was not present in the TMJ articular cartilage as we did not examine it histologically, and we have only studied it from one individual. Future work should examine the TMJ articular cartilage as it has a unique cartilage composition being both fibrocartilaginous and hyaline‐like, with type I collagen and type II collagen (Mizoguchi et al., 1996, Wang et al., 2009, Delatte et al., 2004, Teramoto et al., 2003). AKU mice present the best opportunity to understand the pathology (or lack of) within the TMJ in AKU, in addition to studying other fibrocartilage such as the sternoclavicular and pubic symphysis articular discs which have not previously been examined in mice (Hughes et al., 2021).

### 4.7 Sclera

The sclera is a dense fibrous tissue predominantly formed from type I collagen and small amounts of type III collagen (Keeley et al., 1984), embedded in a proteoglycan matrix. We show the first histology of pigmented sclera showing both diffuse and focal aggregates of pigmentation. Grossly, the pigmentation appears to be deep to the blood vessels situated within the episcleral layer, and therefore is in the thickest, deeper stromal layer, confirmed by histology. The nasal and temporal scleral pigmentation observed here is well reported in AKU, typically located within the palpebral fissure, and often observed in the conjunctiva (Lindner & Bertelmann, 2014; Ranganath et al., 2020). It has previously been suggested that both UV light damage and stress arising from muscle contraction may be damaging factors that lead to scleral/conjunctival pigmentation (Ranganath et al., 2019). Although UV light could be a contributing factor, it would not explain the localisation of pigment to the nasal and temporal sclera only. Stress from the medial and lateral recti muscles may explain the localisation of pigmentation medially and laterally but does not explain the lack of pigmentation where the superior and inferior recti muscles, and the oblique muscles attach. It is possible that the deeper ciliary body muscles could be causing tension on the sclera.

### 4.8 Heterogeneity of pigmentation

The distribution of pigmentation was very heterogenous both across and within the different connective tissues examined, with vast differences in pigment intensity. This heterogeneity is not a new phenomenon; even between siblings carrying the same genetic HGD variants, considerable variability in the AKU disease phenotype has been observed (Vilboux et al., 2009; Zatkova et al., 2022). Heterogeneity of ear cartilage pigmentation is reported, and has been described as localised and diffuse, and not uniform across the tissue (Al‐Shagahin et al., 2019; Ranganath et al., 2020). Zatkova et al. (Zatkova et al., 2022) suggest other factors such as genetic, biomechanical and environmental factors modify the disease, causing accelerated pigmentation and tissue damage in some patients and not others.

Pigmentation clearly favours connective tissues, particularly those that have a high fibrillary collagen content, namely collagen types I and II. In addition to different functions and tissue compositions, tissues also age and degenerate at different rates. The exposed collagen hypothesis of ochronotic pigmentation suggests that pigmentation requires changes in the extracellular matrix to occur (Gallagher et al., 2016), with pigmentation taking weeks to become visible histologically in mice (Hughes et al., 2021; Keenan et al., 2015) and years to become visible externally in humans. Whilst there is some evidence that points towards collagen as the binding site of HGA/pigment (Chow et al., 2011; Taylor et al., 2011), it has not been definitively proven.

### 4.9 Nitisinone

The ochronosis observed in this dissection is much less than that observed by Helliwell et al. (2008); they investigated a donor of 74 years who had not received HGA‐lowering treatment. The individual here was 60 years old and had received HGA‐lowering nitisinone treatment for 7 years prior to death. There could have been some reversal of ochronosis on nitisinone therapy, as previously described by Ranganath et al. (Ranganath et al., 2020), who showed a decrease in ochronosis scores of externally visible eye and ear pigment and histological assessment of ear biopsies. The earlier work in AKU mice however demonstrated that cartilage pigmentation could only be halted with nitisinone treatment, and not reversed (Keenan et al., 2015). We cannot deduce whether the milder ochronotic phenotype that we have observed is due to individual differences (age, lifestyle, etc.) or due to nitisinone treatment through the comparison of just two individuals. More studies are needed to investigate the potential mechanism of pigment removal and must consider factors such as tissue type, location and function; different tissues may respond differently to nitisinone/removal of HGA (i.e. sclera versus articular cartilage).

### 4.10 Limitations

Although this study has provided the opportunity for a detailed examination of AKU tissue, an important limitation is that is of one individual. Whilst some of the key findings confirm previous descriptions of pigment localisation, novel findings have not been confirmed in other AKU individuals. Of these, the lack of TMJ pigmentation is notable, as it could provide key information on how to protect other cartilages from pigmentation if it is protected. Another limitation of this study is that tissues were not fixated promptly after death, which was after several days of refrigeration and a freeze–thaw cycle. Although the morphology of visceral tissues was therefore poor, the bone and cartilage histology was of adequate quality, and the identification of pigment was not affected. The presence or absence of ochronotic pigment is very unlikely to have been affected by a delay in tissue acquisition and fixation, as in our experience it is bound irreversibly to the tissue.

### 4.11 Conclusion

The chemical structure of ochronotic pigment must still be elucidated as reviewed by Ranganath et al. (2019), along with the binding site within tissue extracellular matrix. There is, therefore, merit in identifying the connective tissue niche and microenvironment that pigment favours, as its binding sites to protein may reveal information about its structure and reactivity. Whilst the current manuscript has not investigated pigmentation at the nanoscale level, histological analysis is a step forward. Here we confirm that within osteochondral tissues, CAC is very susceptible to pigmentation and show that perichondrium pigments early in both articular and non‐articular cartilage, and before non‐calcified hyaline cartilage. We also show the heterogeneity of pigmentation both within and between different tissues and suggest that studying the fibrocartilaginous disc of the TMJ may provide insights into factors that are protective against ochronotic pigmentation. Whether or not pigmentation is able to reverse within joint tissue when treated with nitisinone requires further investigation.

## AUTHOR CONTRIBUTIONS

JHH, APB, NPT, CMT, PDR, GC and AP were involved in tissue dissection and analysis. JHH and APB wrote the manuscript. All authors contributed to the critical revision and approval of the manuscript. JAG and LRR conceived the study design.

## CONFLICT OF INTEREST STATEMENTS

The authors have no conflicts of interest to declare.

## Supporting information


Data S1.

## Data Availability

The data that support the findings of this study are available from the corresponding author upon reasonable request.
